# Supplementary material for: Identification of photoperiod-induced specific miRNAs in the adrenal glands of Sunite sheep (Ovis aries)
Source: Front Vet Sci. 2022 Jul 22;9:888207. doi: 10.3389/fvets.2022.888207 (PMC9354845; doi:10.3389/fvets.2022.888207)
Supplement: Supplementary file 1 [file Data_Sheet_1.ZIP › Supplementary file/miRDeep2 file.zip!/miRDeep2 file/Novel_Structure/SPLP42A3/Novel_35.pdf]

Provisional ID	:	NC_019484.2_38753
Score total	:	22
Score for star read(s)	:	3.9
Score for read counts	:	14.4
Score for mfe	:	2.1
Score for randfold	:	1.6
Score for cons. seed	:	
Total read count	:	40
Mature read count	:	36
Loop read count	:	0
Star read count	:	4

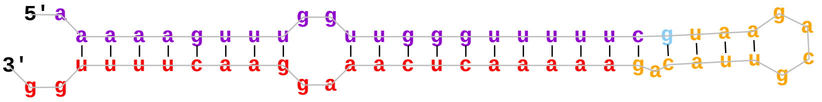

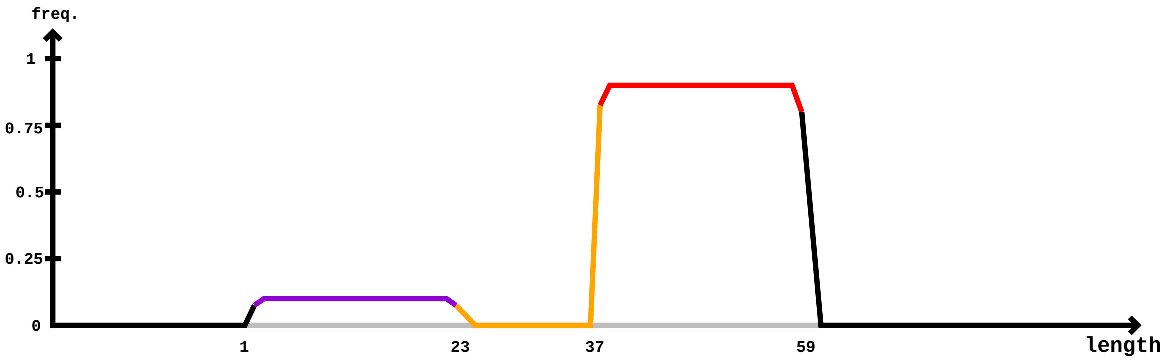


```
                    Star                                   Mature
5'- auaaauuauuggguuggccaaaaaguuugguuggguuuuucguaagacguuacagaaaaacucaaaggaacuuuuggccagcccaauauuauaugauuccaguuauacgaa   -3'    obs
    auaaauuauuggguuggccaaaaaguuugguuggguuuuucguaagacguuacagaaaaacucaaaggaacuuuuggccagcccaauauuauaugauuccaguuauacgaa          exp
    ......(((((((((((((((((.((((..(((((((((((((((....)))).)))))))))))..)))))))))))))))))))))...((((((....)))))).... reads  mm  sample
    ...................aaaaaguuugUuuggguuuuu....................................................................... 1      1   seq
    ...................aaaaaguuugUuuggguuuuuc...................................................................... 2      1   seq
    ....................aaaaguuugguuggguuuuuc...................................................................... 1      0   seq
    .......................................................aaaaacucaaaCgaacuuuug................................... 1      1   seq
    .......................................................aaaaacucaaaUgaacuuuugg.................................. 23     1   seq
    .......................................................aaaaacucaaaUgaacuuuuggc................................. 9      1   seq
    ........................................................aaaacucaaaUgaacuuuug................................... 3      1   seq
```
